# Rates and risk factors of complications associated with operative treatment of pelvic fractures

**DOI:** 10.1007/s00590-022-03375-z

**Published:** 2022-09-04

**Authors:** C. Q. B. Mostert, R. A. Timmer, P. Krijnen, S. A. G. Meylearts, I. B. Schipper

**Affiliations:** 1 Department of Trauma Surgery, Leiden University Medical Centre, Leiden, The Netherlands; 2 Department of Trauma Surgery, Haaglanden Medical Centre, The Hague, The Netherlands

**Keywords:** Pelvic ring fractures, Acetabular fractures, Surgery, Fixation, Post-operative complications

## Abstract

**Purpose:**

Post-operative complications following fixation of pelvic fractures can lead to mortality and increased morbidity. Available literature regarding complications is heterogeneous and knowledge on risk factors is limited. This study aims to identify the most common post-operative complications and their possible risk factors following pelvic fracture surgery.

**Methods:**

A retrospective cohort study was performed in two level-1 trauma centers in the Netherlands between January 2015 and January 2021. Included patients were all adult patients (≥ 18 years) with an operatively treated pelvic fracture (pelvic ring and/or acetabular fractures). Post-operative complications included surgical site infections (SSI), material-related complications, neurological complications, malunion/non-union and performed reoperations. A forward stepwise multivariable logistic regression analysis was used to identify any risk factors associated with these complications.

**Results:**

Complications occurred in 55 (24%) of the 233 included patients. SSI’s were most common, occurring in 34 (15%) patients. Duration of surgery (odds ratio 1.01 per minute, 95% confidence interval 1.00–1.01) and obesity (odds ratio 1.10 per BMI point, 95% confidence interval 1.29–7.52) were independent risk factors for development of SSI. Less common post-operative complications were material-related complications (8%) and neurological damage (5%).

**Conclusion:**

Limiting operation time by using less invasive and less time-consuming surgical approaches may reduce the risk of SSI. More awareness and post-operative screening for early signs of SSI is mandatory, especially in obese patients. Future research should include large prospective patient cohorts to determine risk factors for other post-operative complications associated with pelvic fracture surgery.

## Introduction

Pelvic fractures, including both pelvic ring and acetabular fractures, are often caused by high-energy trauma such as traffic accidents. They occur in up to 25% of severely injured patients [6, 22]. Pelvic fractures can have a profound impact on the patient’s long-term quality of life; many of them do not fully regain their initial level of physical functioning, and experience mental health problems [5] Often surgical fixation of the pelvic ring and/or acetabulum is required to restore stability and improve patient outcomes [23]. Although many surgical techniques and approaches for fixation of pelvic ring and acetabular fractures are described in the literature, open reduction and internal fixation (ORIF) is still the gold standard, since it provides the best possibilities for adequate fracture reduction and fixation, as well as the best long-term results [9, 10].

Pelvic fracture surgery is associated with complication rates up to 35% [12, 16]. These complications include post-operative infections, neurological complications, re-operations, and mortality [3, 4]. Knowledge of post-operative complication rates and risk factors associated with pelvic fracture fixation is needed for developing prevention strategies and improving patient outcomes [11]. Although understanding the risk factors for surgical complications is important, the available literature regarding complications after pelvic fracture surgery is heterogeneous and mostly addresses only specific subgroups of patients, fracture types or surgical techniques [11, 20].

The current study includes a cohort of patients with pelvic ring, acetabular or combined fractures, that were operated on via commonly used surgical approaches. The aim of this study was to investigate the incidence of common surgical complications and identify their potential risk factors.

## Methods and materials

A retrospective cohort study was performed in two level-1 trauma centers in the Netherlands: the Leiden University Medical Center (LUMC) in Leiden and the Haaglanden Medical Center (HMC) in The Hague. The study protocol was approved by the regional Medical Ethics Review Board (protocol nr. G20.118).

### Patients

All adult patients (≥ 18 years old) with a surgically treated pelvic ring and/or acetabular fracture in the period from January 2015 up to January 2021 were selected from the regional trauma registry and included for analysis. Patients with previous pelvic ring or acetabular fractures and patients with pathological fractures were excluded.

All patients received standard of care according to the current hospital’s operative and post-operative treatment guidelines regarding traumatic pelvic fractures. According to these guidelines, all patients received standard antibiotic prophylaxis of 1 or 2 g of cefazolin. All operations were conducted by a senior trauma surgeon trained and experienced in pelvic fracture surgery, assisted either by another trauma surgeon or a surgical resident in training.

### Data collection

All data were collected from electronic patient records of participating centers. Data concerned the number and type of complications including surgical site infections (SSI). SSI were defined if they were indicated as such in the patient records or if there were local signs of infection at the incision site (e.g., redness, wound fluid leakage) either combined with systemic signs of infection (e.g., fever, leukocytosis). Material-related complications were defined as break-out of material and mechanical irritation followed by material removal. Nerve damage was defined as persistent nerve damage after 6 months since surgery*.* Other study parameters included mal- and non-union, reoperation, baseline characteristics including demographic data, obesity defined as body mass index ≥ 30, ASA Physical Status Classification, smoking, osteoporosis, use of anticoagulants, fracture and other injury characteristics including high or low energy trauma, applied treatment, surgical intervention (Kocher- Langenbeck approach, (Modified) Stoppa approach, ilioinguinal approach, percutaneous fixation and external fixation), operation time (time between incision and wound closure), materials used, use of pre- and post-operative antibiotics, and hospital outcomes including duration of hospital stay, ICU admission and in-hospital mortality. Data were stored in a secured and coded database in the Castor Electronic Data Capture (EDC) System.

Pelvic ring fractures were classified according to the Young-Burgess classification and acetabular fractures according to the Letournel classification [1, 18].

### Statistical analysis

Data are presented for the total group and per fracture type. Categorical data are presented as number and percentage, and continuous variables were presented as mean with standard deviation (SD) or as median with interquartile range (IQR) if not normally distributed according to the Shapiro–Wilk test. Differences in the mean operation time between surgical approaches were tested using ANOVA. Forward stepwise multivariable logistic regression analysis (*p*-to-enter = 0.05) was used to identify risk factors for the most prevalent post-operative complication (SSI). All analyses were performed using IBM’s statistical package for social sciences (SPSS) version 27.

## Results

### Baseline characteristics and hospital outcomes

The study population consisted of 233 patients. Baseline characteristics are presented in Table 1. The median age was 59 years (IQR 38–72), and 62% of patients were male. Seventy-seven percent of the patients had sustained high-energy trauma. Twenty-nine percent of the patients were admitted to the ICU. Six patients died during hospital admission (3% of patients). Five patients died due to other injuries than the pelvic fracture. One patient died one week after the initial operation due to a bowel perforation and one patient died because of an in-hospital cardiac arrest (Table 1).

**Table 1:** Patient and injury characteristics and hospital outcomes of patients with pelvic fractures

Variable	All patients *n* = 233	Pelvic ring fracture *n* = 127 (55%)	Acetabular fracture *n* = 101 (43%)	Combined fractures *n* = 5 (2%)
*Patient data*				
Age; median (IQR)	59 (38–72)	56 (34–72)	62 (43–72)	35 (31–50)
Male; *n* (%)	144 (62)	60 (47)	82 (81)	2 (40)
Obese; yes *n* (%)	35 (15)	16 (13)	16 (16)	3 (9)
*Medical history; n (%)*				
Chronic diseases	119 (51)	66 (52)	51 (51)	2 (40)
Previous trauma	19 (8)	9 (7)	9 (9)	1 (20)
Use of anticoagulants; *n* (%)	39 (17)	22 (17)	17 (17)	0 (0)
*ASA physical status; n (%)*				
ASA 1	43 19)	25 (20)	17 (17)	1 (20)
ASA 2	103 (44)	57 (44)	45 (45)	2 (40)
ASA 3	67 (29)	34 (27)	32 (32)	1 (20)
ASA 4	20 (9)	12 (9)	7 (7)	1 (20)
*Smoking; n (%)*				
Non-smoker	152 (65)	77 (60)	74 (73)	1 (20)
Former smoker	22 (9)	14 (11)	8 (8)	0 (0)
Smoker	49 (21)	29 (23)	17 (17)	3 (60)
Osteoporosis; n (%)	33 (14)	23 (18)	10 (10)	0 (0)
*Injury data*				
*Energy trauma; n (%)*				
High energy	180 (77)	95 (75)	80 (79)	5 (100)
Low energy	53 (23)	32 (25)	21 (21)	0 (0)
*Hospital outcomes*				
ICU admission; n (%)	68 (29)	49 (38)	15 (15)	4 (80)
Duration of ICU admission in days; median (IQR)	2 (1–6)	2 (1–6)	1 (1–3)	4 (3–6)
Duration hospital stay in days; median (IQR)	12 (8–20)	12 (7–21)	12 (9–17)	28 (18–84)
In-hospital mortality; n (%)	6 (3)	5 (4)	1 (1)	0 (0)

*IQR* Interquartile range; *BMI* Body mass index; *ASA score* American Society of Anethesiology physical status score; *ICU* intensive care unit

### Pelvic ring fractures

Pelvic ring fractures were diagnosed in 127 (55%) patients. Most common were Lateral Compression (LC) type 1 fractures (*n* = 37; 29%), LC type 2 fractures (*n* = 22; 17%) and isolated sacrum fractures (*n* = 19; 15%). (Table 2) A percutaneous surgical approach was used in 39 patients (31%) and the (Modified) Stoppa approach in 35 patients (28%). Twenty-eight patients were treated using external fixation, of whom 22 received internal fixation and 6 external fixation as definite treatment.

**Table 2:** Fracture classification, surgical approaches and fixation materials, by fracture type

	Pelvic ring fracture	Acetabular fracture	Combined fracture
	*n* = 127 (55%)	*n* = 101 (43%)	*n* = 5 (2%)
*Young and burgess classification; n (% of total)*			
LC I	37 (29)		4 (80)
LC II	22 (17)		0 (0)
LC III	8 (6)		0 (0)
APC I	8 (6)		0 (0)
APC II	11 (9)		0 (0)
APC III	8 (6)		0 (0)
Vertical shear	12 (9)		1 (20)
Combined	2 (2)		0 (0)
Isolated sacrum fracture	19 (15)		0 (0)
*Letournel classification: n (% of total)*			
Posterior wall		11 (11)	0 (0)
Posterior column		1 (1)	0 (0)
Anterior wall		2 (2)	0 (0)
Anterior column		15 (15)	0 (0)
Transverse		3 (3)	1 (20)
Posterior column & wall		5 (5)	0 (0.0)
Transverse posterior wall		4 (4)	1 (1)
T-shaped		4 (4)	0 (0)
Anterior, posterior hemi-transverse		27 (27)	2 (40)
Both columns		29 (29)	1 (20)
*Surgical approach; n (% of total)*			
Modified stoppa	35 (28)	51 (51)	2 (40)
Ilioinguinal	2 (2)	6 (6)	0 (0)
Kocher-Langenbeck	1 (1)	23 (23)	0 (0)
Percutaneous	39 (31)	0 (0)	0 (0)
Midline lumbosacral incision	7 (6)		
Modified Stoppa + Ilioinguinal lateral window	4 (3)	5 (5)	1 (20)
Modified Stoppa + Kocher- Langenbeck	0 (0)	9 (9)	0 (0.0)
Modified Stoppa + Percutaneous	21 (17)	1 (1)	0 (0)
Modified Stoppa + Medial lumbosacral incision	2 (2)	0 (0)	1 (1)
Ilioinguinal + Percutaneous	1 (1)	0 (0)	0 (0)
Total external fixator placed	28 (22)	4 (4)	0 (0)
External fixator as definite treatment	6 (5)	0 (0)	0 (0)
Other approaches*	15 (12)	6** (6)	1*** (20)
*Fixation material; n (% of total)*			
Plate	57 (45)	96 (95)	5 (100)
Screws	93 (73)	27 (27)	3 (60)
Anterior screw fixation	30 (23)	15 (15)	2 (40)
Posterior screw fixation	1 (10)	7 (7)	0 (0)
SI-screw fixation	74 (58)	6 (6)	1 (20)
Pedicle screw fixation	6 (5)	0 (0)	0 (0)
Primary total hip prothesis	0 (0)	5 (5)	2 (9.1)
Secondary placement of THP	0 (0)	8 (8)	0 (0)

*Other surgical approach pelvic ring fractures; Pfannenstiel incision *n* = 5; Incision near the SI joint *n* = 2; Smith-Peterson approach *n* = 1; Iliofemoral approach *n* = 1; External fixator only *n* = 6

**Other surgical approach acetabular fractures; Lateral approach *n* = 1; Parasymphysisis approach *n* = 1; Smith-Peterson approach *n* = 1; Iliofemoral approach *n* = 1; Modified Gibson approach *n* = 1; Median laparotomy *n* = 1

***Other surgical approach combined fractures; Prolonged abdominal incision followed by a modified stoppa *n* = 1

### Acetabular fractures

In total, 101 (43%) patients suffered from an acetabular fracture. Both column type was seen in 29 patients (29%) and anterior posterior hemi-transverse fracture in 27 patients (27%). (Table 2) The (Modified) Stoppa approach was used in 51% of patients (*n* = 51) and the Kocher-Langenbeck was used in 23% of patients (*n* = 23). Five patients received a total hip prosthesis as a primary treatment without previous fixation (5%). Secondary hip protheses were placed in 8 (8%) patients.

### Combined fractures

Five patients (2% of the cohort) with median age of 35 years (31–50) sustained combined pelvic ring and acetabular fractures. All of these patients sustained a high energy trauma. The modified Stoppa approach was used in 2 patients (40%), two patients were treated with a (modified) Stoppa combined with an ilioinguinal approach, medial lumbosacral incision or a prolonged abdominal incision. (Table 2).

### Duration of surgery

The mean duration of surgery differed significantly between the surgical approaches (*p* < 0.01). The shortest operation time was recorded for patients undergoing surgical fixation via the percutaneous approach (mean 57 min; 28 SD). The longest duration of surgery was recorded for the group in which a combination of the modified Stoppa and Kocher-Langenbeck approach was used (mean 277 min, SD 50) (Table 3).

**Table 3:** Mean duration of pelvic fracture surgery, by surgical approach

Operative approach	Mean duration in minutes (SD)	*p* (ANOVA)
Modified Stoppa	148 (60)	< 0.01
Ilioinguinal	204 (58)	
Kocher-Langenbeck	161 (62)	
Percutaneous	57 (28)	
Modified Stoppa + ilioinguinal	228 (64)	
Modified Stoppa + KL	277 (50)	
Modified Stoppa + percutaneous	146 (60)	

### Post-operative complications

Seventy post-operative complications occurred in 50 (21%) patients (Table 4). SSI was observed in 34 patients of which 29 subsequently needed some sort of re-operation (mainly operative debridement because of SSI). Osteomyelitis in addition to a deep infection was diagnosed twice. (Table 4) Overall, Staphylococcus Aureus was found in six percent of patients with an infection, and another 6% had an isolated staphylococcus epidermidis in their wound culture. In 5% an Enterococcus faecium and in 5% Streptococcus anginosus was found in the wound cultures. Ten percent of the patients witch an infection was treated with Flucloxacillin, and 7% was treated with Vancomycin. Material-related complications occurred in 14 patients. Irritation due to fixation material subsequently led to osteosynthesis removal in 8 cases and plate breakage in 6 cases. Impaired fracture healing, including non-union and malunion, was diagnosed in 8 patients and eventually led to revision surgery in 6 cases. Persistent neurological complications occurred in 10 patients (Table 4).

**Table 4:** Post-operative complications in 55 patients with pelvic fractures

	Number of complications	Number of re-operations due to complication
Total	70	
Infection	34	29
Osteomyelitis	2	1
Material related complications	14	11
Plate breakage	6	3
Mechanical irritation with material removal	8	8
Persistent neurological deficit	10	1
Non-union	7	5
Malunion	1	1
Other*	1	1

*One patient developed a paralytic ileus followed by a bowel perforation a week after initial operation. The patient needed acute surgery to repair the bowel and to shorten the acetabular screw. Complication possible the result of the pelvic surgery or due to the initial injury

### Risk factors for surgical site infections (SSI)

Univariable analysis showed that patients with SSI were significantly younger (mean 56 years vs. 50 years; *p* = 0.09; Table 5), more often suffered from obesity (35% vs 12%; *p* = 0.001), had a significantly longer duration of surgery (179 min vs 138 min; *p* = 0.003) and hospital stay (28 days vs 17 days; p = 0.08). The use of external fixator before definitive surgery, the amount of ICU admission, smoking and high or low-energy trauma did not differ significantly between the patients with or without SSIs. (Table 5) In the multivariable logistic regression analysis using forward selection, presence of obesity (OR: 3.12; 95% CI: 1.29–7.52; p = 0.01) and duration of surgery (OR per minute increase: 1.01; 95% CI: 1.00–1.01; *p* = 0.04) were identified as independent risk factors for the development of SSI (Table 5).

**Table 5:** Univariable association of patient- and fracture-related characteristics with post-operative infection in patients with pelvic fractures

Variable	No surgical site infection (*n* = 199)	Surgical site infection (*n* = 34)	*p*-value
Age; mean (SD)	56 (20)	50 (18)	0.09
Obese; *n* (%)	23 (12)	12 (35)	0.001
Duration of surgery in minutes; mean (SD)	138 (74)	179 (71)	0.003
ICU admission; *n* (%)	58 (29)	10 (29)	0.98
Smoking; *n* (%)			0.19
Non-smoker; n (%)	153 (79)	21 (68)	
Smoker; *n* (%)	42 (21)	10 (32)	
Energy trauma; *n* (%)			0.91
High energy	154 (77)	26 (76)	
Low energy	45 (23)	8 (24)	
External fixator before definitive surgery; *n* (%)			
No fix ex; *n* (%)	179 (90)	28 (82)	0.19
Fix ex; *n* (%)	20 (10)	6 (18)	
Surgical approach; *n* (%)			
Modified Stoppa	77 (39)	11 (32)	0.12
Kocher-Langenbeck	21 (11)	2 (6)	
Percutaneous	36 (18)	3 (9)	
Other*	65 (33)	18 (53)	

*All other approaches as described in Table 2

## Discussion

This cohort study found an overall complication rate following operative treatment of pelvic ring and acetabular fractures of 21% and in-hospital mortality of 3%. The most common post-operative complication was SSI (accounting for 49% of all reported complications) followed by material-related (20%) and neurological complications (14%). Multivariable analysis identified duration of surgery and obesity as independent risk factors for SSI’s.

### Surgical site infections (SSI)

In the current study, patients’ age, obesity, duration of surgery and hospital stay were significantly associated with SSI in the univariable analysis (Table 5). In the multivariable analysis, obesity (OR 3.12; *p* = 0.01) and duration of surgery (OR: 1.006; *p* = 0.04) remained independent, statistically significant predictors for the development of SSI after pelvic fracture surgery. (Table 3) In pelvic fracture surgery, ORIF is still the gold standard since it provides excellent fracture exposure. The downside of open surgery, however, is the prolonged operation time and large surgical wound areas which increase the risk of SSI as shown by our study and other literature [8]. Based on these findings avoiding prolonged operation times seems of key importance to reduce the risk of SSI. In the current study, the shortest operation time (57 min; 28 SD) was recorded in patients treated via the percutaneous approach. This approach is not only associated with shorter operation times but because of their minimally invasive nature also involve smaller surgical wound areas. However, for complex multi-fragment displaced pelvic ring or acetabular fractures, a percutaneous approach is not possible because of technical reasons including unsatisfactory fracture exposure leading to inadequate fracture reposition or fixation. Still further development of novel minimally invasive and efficient operation techniques is key for reducing operation time and wound exposure which would reduce the risk of post-operative infections [2].

Another significant risk factor identified in the current study was obesity. Several underlying causes such as lower tissue levels of prophylactic antibiotic and also prolonged operation times are hypothesized to lead to the increased risk of SSI in obese patients [7, 13, 21]. Extra pre- and post-operative attention, adequate pre-operative counseling about the increased risk of SSI should be considered in these patients. Furthermore, extra attention and awareness of early signs of SSI is recommended to prevent or, if present, to detect SSI in a early stage. Early detection followed by adequate treatment leads to less severe outcomes and could prevent the necessity of operative debridement [25, 26]. However, in the current study, almost all patients with SSI needed revision surgery which can lead to a significantly longer hospital stay, adverse outcomes or higher healthcare costs.

### Neurological complications

Persistent neurological complications (13% of all complications) were relatively uncommon which is in accordance with existing literature [15]. Neurological complications can either be caused by the accident itself or be induced by the intervention. In acetabular fractures involvement of the posterior wall and the Kocher-Langenbeck approach are more commonly associated with nerve damage since especially the sciatic nerve is at risk [17]. This is also the case for posterior fixation of pelvic ring fractures [15]. However, when anterior pelvic ring fractures are fixated using for example the (modified) Stoppa approach, the neurovascular bundles of the bladder are particularly at risk [15]. Also, iatrogenic injury of the obturator and sciatic nerve has been described when using this approach [15].

### Material-related complications

Although less common, material-related complications after acetabular and pelvic ring surgery are reported and associated with severe adverse outcomes including chronic residual pain, material removal, deformity, and progressive physical impairment [19, 24]. Material-related complications including plate breakage and mechanical irritation accounted for 13% of all complications observed in the current study. Literature reporting on material-related complications after acetabular or pelvic ring treatment reports complication rates between 0 and 22% but is heterogeneous with respect to definition and included patients [3, 15].

### Impaired fracture healing

Although malunion and non-union of surgically treated acetabular and pelvic ring fractures are rare, treating these conditions can be very challenging [14]. Since mal and non-union are not common, literature regarding this matter is scarce and exact incidence rates are not clearly defined. This is partly due to the differences in the definition of mal and non-union between studies [14]. In the current study, impaired fracture healing diagnosed on radiographs occurred in 3% of the patients. Several underlying causes such as low-grade infections, osteoporosis or other conditions involving bone demineralization are associated with the development of non- and malunions. However, most cases of mal-union or non-union after pelvic fracture surgery are caused by delayed or inadequate fixation [27]. Frequent follow-up using physical examination and radiographic evaluation is necessary to detect this complication and to treat it accordingly.

### Study limitations

The main limitations of the study are related to the retrospective design of the study and its small sample size. Therefore, the statistical power was limited, and only two independent risk factors for the most common complication (SSI) could be identified in the multivariable analysis. Due to the retrospective study design and without data on follow-up after hospital discharge, it was not possible to assess the effects of complications on long-term functional outcomes.

## Conclusion

The most common post-operative complications after surgery of pelvic ring or acetabular fractures are SSI’s, which account for almost half of the reported complications. Independent risk factors for SSI’s were increased duration of surgery and obesity. These findings stress the need to avoid prolonged operation times and encourage the use of minimally invasive surgical approaches. Furthermore, extra post-operative awareness of early signs of SSI is recommended to prevent adverse outcomes and re-operations, especially in obese patients since these patients are highly susceptible for developing SSI’s. More prospective research is needed with larger patient cohorts including all subgroups of patients, fracture types and surgical techniques to further determine risk factors for post-operative complications associated with pelvic fracture surgery.
